# Effect of Catalase and Sodium Fluoride on Human Enamel bleached with 35% Carbamide Peroxide

**DOI:** 10.5005/jp-journals-10005-1276

**Published:** 2015-04-28

**Authors:** Ruchi Thakur, Anand L Shigli, Divya S Sharma, Gagan Thakur

**Affiliations:** Senior Lecturer, Department of Pedodontics, People’s College of Dental Sciences and Research Centre, Bhopal, Madhya Pradesh India; Professor and Head, Department of Pedodontics and Preventive Dentistry, Bharati Vidyapeeth University Dental College and Hospital, Sangli Maharashtra, India; Professor and Head, Department of Pedodontics and Preventive Dentistry, Modern Dental College and Research Centre, Indore, Madhya Pradesh India; Reader, Department of Oral and Maxillofacial Surgery, People’s College of Dental Sciences and Research Centre, Bhopal Madhya Pradesh, India

**Keywords:** In-office bleaching, Carbamide peroxide, Catalase, Sodium fluoride.

## Abstract

**Aim:** To evaluate the effects of postbleaching antioxidant application fluoridation treatment on the surface morphology and microhardness of human enamel.

**Materials and methods:** Ten freshly extracted human maxillary central incisors were cut at cementoenamel junction. Crown portion was sectioned into six slabs which were divided into five groups: group A – untreated controls; group B – 35% carbamide peroxide (CP); group C – 35% CP and catalase; group D – treatment with 35% CP and 5% sodium fluoride; group E – 35% CP, catalase and 5% sodium fluoride. Thirty-five percent carbamide peroxide application included two applications of 30 minutes each at a 5-day interval. After treatment, the slabs were thoroughly washed with water for 10 seconds and stored in artificial saliva at 37°C until the next treatment. Two percent sodium fluoride included application for 5 minutes. Three catalase included application for 3 minutes.

**Results:** After 5 days, groups B and C showed significantly decreased enamel microhardness compared to control. Group D specimens showed relatively less reduction in enamel micro-hardness than group C specimens. There is a marked increase in enamel microhardness in group E specimens.

**Conclusions:** Fluoride take up was comparatively enhanced after catalase application resulting in less demineralization and increased microhardness.

**How to cite this article:** Thakur R, Shigli AL, Sharma DS, Thakur G. Effect of Catalase and Sodium Fluoride on Human

Enamel bleached with 35% Carbamide Peroxide. Int J Clin Pediatr Dent 2015;8(1):12-17.

## INTRODUCTION

Bleaching provides more conservative whitening than other modalities like microabrasion and other restorative procedures. However, high concentration peroxides have showed potential adverse effects in animal and human studies ranging from tooth sensitivity, alteration in surface morphology of enamel, reduced bond strength of resin materials to carcinogenicity.^1,2^

Agents mainly used for dental bleaching are different concentrations of carbamide or hydrogen peroxide (HP). Carbamide peroxide (CP) eventually dissociates into urea and hydrogen peroxide. Hydrogen peroxide ionization occurs in the presence of decomposition catalysts, enzymes and saliva producing free radicals like nascent oxygen (O)–weaker free radical), hydroxyl or perhydroxyl ions (HO_2_)–more potent free radical). Free radicals are deficient electrons and their neutralization process results in oxidation of the neutralizing agent. In commercially available pure aqueous form, HP is weakly acidic to extend shelf life. However, to promote the formation of perhydroxyl, HP needs to be made alkaline (pH 9.5-10.8).^3,4^ Free radicals are able to react with the electron rich regions of pigments inside the dental tissues, by diffusing through the interpris-matic substance (organic content), breaking down large pigmented molecules into smaller, less pigmented ones. Higher concentration of peroxides produces whitening results faster abundant production of free radicals. Excessive and prolonged presence of free radicals in dental tissues can lead to a continued oxidation process. Active detoxication in human body is based on three enzymes composing the basis of antioxidant defence system–superoxide dismutase, catalase and glutathione peroxides, which can be found in cells where the oxidative stress is greater like the cytosol, mitochondria and peroxy-somes.^5^ Role of catalase is to prevent this overproduction of free radicals by inactivating the molecules responsible for their production. It accelerates transformation of HP into water and oxygen hence, acts as a neutralizing agent causing removal of residual and unwanted free radicals. Catalase is a tetramer of four polypeptide chains, each over 500 amino acid long. It contains four porphyrin heme (iron) groups that allow the enzyme to react with HP. The optimum pH for catalase is approximately seven (6.8-7.5). Oxygen in the gaseous form is evaporated and water provides necessary hydration to the enamel surface required after bleaching. The amount of peroxide decomposed is directly proportional to the concentration of catalase. Rate of this reaction is rapid and little energy is involved as catalase accelerates the decomposition of HP by 100,000,000 fold.^6^ Jones and Wynne-Jones^7^ have proposed that the catalytic action of Fe^3+^ ion both in the decomposition of HP and as a mediator of coupled ‘per-oxidatic’ oxidations. The metal acts as a redox catalyst of the catalyst reaction through three reactions:

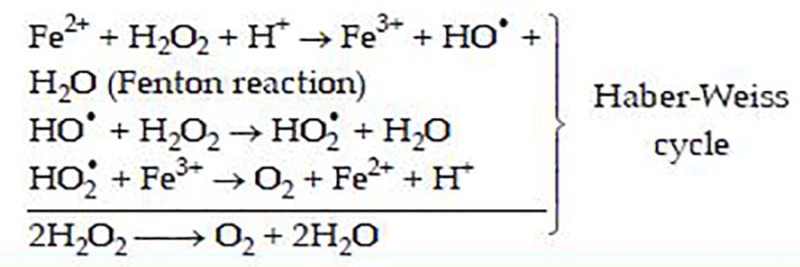



Evident microstructural changes in bleached enamel have been seen ranging from mild, moderate to severe^8^ and loss of calcium.^9,10^ These changes can mainly be attributed to oxidative process involved and low pH of the bleaching agent. Possible risk factors and causes of tooth sensitivity may include the patient’s inherent sensitivity, pH and concentration and frequency of active bleaching ingredient.^11^ Tooth sensitivity has also been attributed to penetration of HP into the pulp chamber.^12^ Water based formulations to prevent desiccation of dental tissues and addition of potassium fluoride and/or fluoride to various bleaching agents aids in reducing postbleaching senstivity.^13^

However, free radicals produced by bleaching agent interact with fluoride present in the gel and reduce enamel fluoride acquisition or retention. Therefore, this present study through Vicker’s microhardness changes and scanning electron microscop (SEM) hypotheses that cata-lase application postbleaching ensures a greater uptake of fluoride ions and results in increased microhardness.

**Table 1:** **Table 1:** Description of experiment groups

*Groups*		*Description of experimental groups*	
Control group 35%		A Slab untreated and stored in artificial saliva	
Carbamide peroxide		B Slab treated with 35% CP and stored in artificial saliva	
		C Slab treated with 35% CP, treated with catalase and stored in artificial saliva	
		D Slab treated with 35% CP, treated with 5% sodium fluoride and stored in artificial saliva	
		E Slab treated with 35% CP, treated with catalase, treated with 5% sodium fluoride and stored in artificial saliva	

CP: Carbamide peroxide

### Specimen Preparation

**Figs 1A to E:**
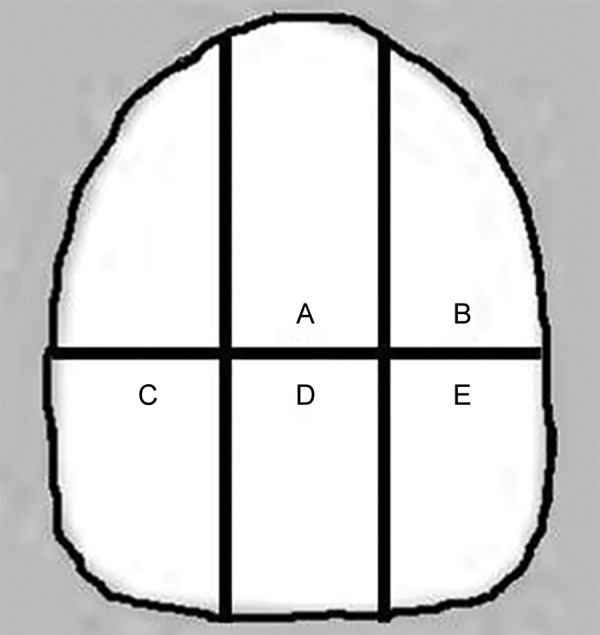
Extracted incisor crowns were sectioned as per markings shown in the figure to obtain six enamel slabs (approximately 3.5 × 2.5 mm^2^ in dimension) from each central incisors

Thirty freshly extracted human central incisors stored in 0.1% buffered thymol solution (pH = 7) for no longer than 4 weeks after extraction were used in this study. The teeth were cleaned of gross debris and examined under magnification (20×) and 4 incisors, thereby were discarded due to stains, microcracks and/or surface defects. For the remaining 26 incisors, the roots were removed at the cementoenamel junction and the crowns were further sectioned with doublefaced diamond disks using a low-speed handpiece as shown in the figure (Figs 1A to E) to obtain 6 enamel slabs (approximately 3.5 × 2.5 mm^2^ in dimension) from each central incisors. A total number of 156 enamel slabs were thus, obtained (26 incisors × 6 sectioned enamel slabs = 156 enamel slabs). Five slabs (coinciding to following 5 groups–A, B, C, D and E) from each incisor were randomly designated to each group while remaining 1 enamel slab (1 slab × 26 incisors = 26) from each incisor was discarded. Therefore, a final sample size of (n = 130) was equally divided among the following five groups such that each group comprised of 26 slabs each.

### Experimental Groups (Table 1)

Experimental design was as follows (Table 1):

- Control group–A
- Experimental groups–B, C, D and E.

Before placing these slabs in separate prelabelled plastic vials apart from the labial enamel surface, all others were coated with dental varnish to make these surfaces impermeable. The uncoated labial enamel surface was sequentially polished by means of 400, 600, 1200 grade sandpaper. These slabs were subjected to steam sterilization to avoid bacterial contamination and thereafter, immersed and stored in deionized water at 37°C in the respective vials till the commencement of the study.

As per the treatment design stated in Table 1, agents were applied on slabs as follows:

- Regimen for 35% carbamide peroxide [(Gel form available in syringe)–Vishal Dentocare Pvt Ltd] application included two applications of 15 minutes each at a 7-day interval. After treatment the slabs were thoroughly washed with deionized water for 10 seconds and stored in artificial saliva (pH 7.0–Pure Pharma Ltd) at 37°C which shall be changed every 24 hours, until the next treatment.
- Regimen for sodium fluoride [(sodium fluoride I P– 50 mg (equivalent to 2,2600 ppm of fluoride) – Fluoritop-SR^®^ Varnish: slow release topical dental fluoride– ICPA Health Products Ltd)] – One drop was taken each time and using an applicator brush, varnish was painted on the tooth surface.
- Regimen for catalase (enzymatic activity units/mg protein 2000.00-5000.00 Hi–Media Labs Pvt Ltd) included application for 3 minutes. Later was washed with deionized water and placed in respective vial containing artificial saliva.

### Microhardness Testing

Microhardness measurements were made for control slabs (without any treatment so was considered as the baseline value of VHN) and experiment slabs after respective treatment. Slabs were positioned perpendicularly to the long axis of the indentor to record the Vicker’s hardness number (VHN). The individual testing of the enamel slabs was blinded and a 1 kg (max) load Vicker’s indentor was attached to a microhardness tester (Leica, VMHT 30 A, M/S Leica, Germany) which performed three measurements in 5 seconds to determine the VHN of each slab.

**Table 2:** **Table 2:** Values of microhardness (VHN) for control and each experimental group

		*Group A* * (enamel slab 1)*		*Group B* * (enamel slab 2)*		*Group C* *enamel slab 3*		*Group D* *enamel slab 4*		*Group E* *enamel slab 5*	
High		244.7		243.4		249.2		268.6		275.1	
Low		216.3		205.4		210.3		245.3		211.1	
Mean (SD)		231.05 (±11.3 )		228.79 (±15.1)		231.43 (±15.2)		256.84 (± 6.77)		263.74 (±18. 9)	
Median		231.7		236.2		238.4		259.2		269.6	

### Scanning Electron Microscope

The individual testing of the enamel slabs was blinded and enamel surfaces were sputter coated with gold in a vacuum evaporator and photomicrographs of representative areas were taken at 2000× magnifications.

## RESULTS

One way ANOVA test on intergroup comparison was applied which disclosed difference in hardness values both among different experimental groups and control group and suggested a highly significant p-value (p ≤ 0.0001). It is evident from the table that hardness values (Table 2) have decreased significantly in group B as compared to group A and increased significantly in group E as compared to group A.

**Figs 2A to E:**
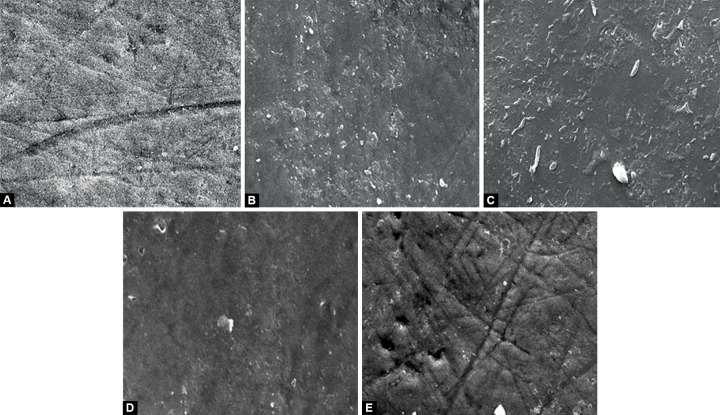
After 5-day interval bleaching, antioxidant and remineralization treatment period, the specimens showed different surface morphology: (A) Unaltered enamel surface, (B) an eroded pattern was noted with narrow cracks and granular material in some areas, (C) thin fibrillar network pattern formed a covering of the entire surface hence obscuring the underlying mild surface alterations, (D) widespread erosions and a mutilayered appearance due to formation of globular structures and aggregations was seen and (E) a relatively smooth surface with tiny depressions all over

After 5 days, specimens showed different surface morphology (Figs 2A to E). The enamel surface was unchanged on the unbleached specimen in group A. An eroded pattern was noted on the specimens in group B with narrow cracks and granular material in some areas. A thin fibrillar network pattern forms a covering of the entire surface hence, obscuring the underlying mild surface alterations in group C. Group D showed a corroded surface morphology with widespread erosions and a mutilayered appearance due to formation of globular structures and aggregations on the enamel surface. Group E shows a relatively smooth surface with tiny depressions all over. The surface morphologic changes in groups B and C were less distinct than those of groups D and E.

## DISCUSSION

The greatest concern since whitening treatments began to be used is the damage that bleaching agents cause to dental tissues, particularly enamel. In confirmation with most similar studies reported in literature, detrimental demineralization and reduced microhardness of enamel was noted in the present study. The present study also evaluates the bleached enamel surface treated with cata-lase and topical fluoride.

Thirty-five percent CP brought about minor alterations in enamel surface in the form of expansion of the prism sheath and narrow gaps resembling cracks between crystals. These gaps were consistent with the organic components in enamel. Mc Guckin^14^ attributed to HP for an increase in porosity as well as extensive fattening in enamel describing an enamel pattern similar to type II etching pattern. Bitter^15^ underlined the importance of considering the effects of these changes on enamel integrity since, in long term, they would be the cause of abrasion or cusp fracture, particularly in teeth that have been restored or weakened by other dental treatment. Further, Lopes^16^ established *in vitro* that erosions in the enamel surface following dental bleaching did not produce a uniform pattern and their intensity varied depending on the sample. There was also an apparent increase in superficial porosity characterised by a greater quantity of tomes process pits that were observed on the samples. It is speculated that it is this factor that explains postbleaching tooth sensitivity. Indeed most bleaching agents are acidic, which is not favorable to enamel, dentin and cementum. Remineralization of bleached enamel is a conventional modality to cure post bleaching hypersensi-tivity. Artificial saliva was used in this study to simulate oral conditions however, its remineralizing potential is not equivalent to natural saliva *in vivo*. Fewer alterations are expected *in vivo*, due to buffer capacity of saliva. It is difficult to determine the clinical significance of the results from *in vitro* studies since calcium and phosphates available in saliva can potentially replenish the lost substance.^17^ When pH is under physiologic limit, part of the phosphorus and calcium particles are released and added to the ionic calcium and phosphorus reservoirs. Consequently, apatite from the enamel surface is protected against dissolution. However, one may recommend the use of artificial saliva consisting only inorganic calcium and phosphate components and devoid of any organic components which could have the potential of forming a protective salivary pellicle. Remineralization by saliva alone may not always be complete, leaving areas susceptible to further decalcification and plaque retention. Therefore, sodium fluoride a well known rehardening (remineralizing) agent. This remineralization process can be explained by fluoride incorporation to tooth structure, forming a calcium fluoride layer that increases hardness values. Fluoride can be weakly or strongly adsorbed at the crystal surface. If it is incorporated into the concentrated layer of ions in the immediate fluid surrounding the crystal, the fluoride is strongly held by the underlying calciumions during an acid challenge. Hence, fluoride at the crystal surface is important, whereas the composition of the underlying apatite will be relatively unimportant. It is also possible for the fluoride to replace hydroxyl ions in the crystal surface and be strongly chemisorbed. In this case, it gets extremely difficult for dissolution of that area of the crystal. An enamel crystal dissolves particularly if fluoride is incorporated in it or on it, this will release fluoride into the external fluid environment. The fluoride in solution will subsequently slow down demineralization. In this way, structurally incorporated fluoride can affect the demineralization process, provided the acid challenge is sufficient to dissolve some apatite with fluoride, but not so strong that only dissolution occurs. This will occur if fluoride is applied frequently to the tooth surface at concentrations low enough to diffuse into the enamel and also if there is a reasonably soluble fluoride form present either at the surface or in the subsurface of the enamel. Fluoride presents in the intercrystalline fluid will enhance this process dramatically. Normal levels of fluoride in whole saliva vary between approximately 0.01 and 0.05 ppm. Studies have indicated that levels as low as 0.1 ppm fuo-ride may be sufficient to enhance crystal growth.

Fluoride in topical form has also been used. The proposed mechanism of action is the occlusion of dentinal tubules by fluoride precipitates. Calcium fluoride and fluorapatite are the major products deposited on the enamel and dentin surfaces after their exposure to high- and low-concentration topical fluoride vehicles respectively.^18^

Tooth sensitivity and reduced bond strength have also been attributed to deeper penetration of HP into dental tissues.^19,20^ Reduction in bond strength is due to the residual oxygen, released from the bleaching agent, that interferes with the resin infiltration into the etched enamel or inhibits the polymerization of resin.^21^ Pre-treatment with antioxidant agent allows free radical polymerization of the adhesive resin to proceed without premature termination by restoring the altered redox potential of the oxidized bonding substrate thus, reversing the compromised bonding.^22^ Also in presence of peroxide, it has been shown that hydroxyl radicals in the apatite lattice are substituted by peroxide ions and produce peroxide-apatite. When peroxide ions decompose, substituted hydroxyl radicals re-enter the apatite lattice, resulting in elimination of the structural changes caused by the incorporation of peroxide ions.^5^ Rotstein^23^ investigated the efficacy of catalase following intracoronal bleaching and observed the total elimination of HP into the extraradicular medium after 3 minutes. This suggests that catalase also provides an appreciably short working time, thereby reducing chairside time.

It is speculated that reducing the residual HP (by the action of antioxidant agent–catalase) could reduce tooth sensitivity after first bleaching application. After final bleaching cycle, removal of HP would facilitate more fluoride uptake on the enamel surface. Carbamide peroxide also infuences enamel fluoride uptake. The reason being, in a wet or humid environment, CP is hydrolytically cleaved, thereby releasing peroxide.^24^ Peroxide will further decompose into radicals which act as the active bleaching agent. It might be speculated that these radicals interact with the fluoride of the gels or the enamel apatite reducing enamel fluoride acquisition or retention. Therefore, for fuoridation of the bleached enamel, instead of using a fuoridated bleaching agent, it could better be performed by a fluoride gel applied after bleaching. Specimens in group D showed a smoothened surface apart from some areas which give an eroded appearance. These eroded areas were areas where further remineralization was still needed, by either artificial saliva or sodium fluoride. Specimens bleached with 38% HP and then treated with Sodium fluoride appear to be comparatively much smoother than their CP counterpart.

Specimens bleached in group E and showed a smooth surface which gives an appearance somewhat similar to that of the control specimen. Tiny depressions of bubbles can be seen on the surface secondary to release of oxygen gas. These might be areas where a neutralizing reaction is still going on because of deeply penetrated HP and subsequently catalase.

## CONCLUSION

Several conclusions have been drawn which highlight the effectiveness of usage of an antioxidant and a hardening agent post bleaching in order to restore the integrity of enamel in terms of remineralization, surface smoothness, hardness and surface hydration. Thirty-five percent CP produced irregularities on the enamel surface which was effectively remineralized by the additive and synergistic effect of catalase and sodium fluoride.
